# Evaluating the Efficacy of a Small Interfering Ribonucleic Acid Molecule, Givosiran, in Treating Acute Intermittent Porphyria: A Systematic Review

**DOI:** 10.7759/cureus.40585

**Published:** 2023-06-18

**Authors:** Priyansh Patel, Sidharth Midha, Surmai Shukla, Divyanshu Dhamija, Adedamola O Bello, Safeera Khan

**Affiliations:** 1 Internal Medicine, California Institute of Behavioral Neurosciences & Psychology, Fairfield, USA; 2 Internal Medicine, Medical College Baroda, Baroda, IND; 3 Radiology, Bharati Vidyapeeth University, Pune, IND; 4 Medicine, California Institute of Behavioral Neurosciences & Psychology, Fairfield, USA; 5 Medicine and Surgery, Qingdao University College of Medical Science, Qingdao, CHN; 6 Medicine and Surgery, California Institute of Behavioral Neurosciences & Psychology, Fairfield, USA; 7 Medicine, Government Medical College Amritsar, Amritsar, IND; 8 Psychiatry, St. Martinus University, Pontiac, USA; 9 Psychiatry, California Institute of Behavioral Neurosciences & Psychology, Fairfield, USA

**Keywords:** porphyria and disability, acute porphyria management, givosiran, sirna, small interfering rna

## Abstract

Acute intermittent porphyria (AIP) is a severe multiorgan dysfunction disorder that can be fatal if not treated promptly. The newest treatment modality involving small interfering RNA (siRNA) molecules, givosiran, is administered for AIP. Although it has very beneficial effects in treating attacks of AIP, it comes with an extensive side effect profile that is not fully understood or studied. Hence, this novel drug model treatment's risk-benefit evaluation is still necessary. For relevant medical literature, we explored medical databases such as PubMed/Medline, PubMed Central, Cochrane Library, Internet Archive Scholar, Google Scholar, and Wiley Online Library. The selected papers were screened based on eligibility criteria and filtered through quality appraisal tools, and 13 finalized research papers were included in the study. Of the 13 identified papers, three were clinical trials, and 10 were review articles. The selected papers all discussed the effectiveness and side effects of givosiran in acute and recurrent attacks of AIP. The research papers showed decreased rates of acute attacks of AIP with givosiran and terminating recurrent attacks. But there are certain non-serious side effects, like fatigue and nausea. Also, there are some severe side effects, like pain. There is limited information on renal and liver function impairment using givosiran and the use of givosiran in patients with kidney and liver disease, for which further studies are required.

## Introduction and background

Porphyria constitutes a group of eight metabolic disorders of the heme e synthesis pathway that can lead to neurovisceral manifestations, dermatological manifestations, or both. Each of these porphyria results in a defect in one or more enzymes of the heme synthesis pathway [1]. Of these, acute intermittent porphyria (AIP) is one of the most common acute porphyrias, with a disease occurrence rate of one in every 20,000 people. AIP is an autosomal dominant disorder caused by a deficiency of the enzyme porphobilinogen deaminase, which converts porphobilinogen to hydroxymethylbilane and is an important step in heme synthesis [2]. The disease severity can be attributed to the activity of hydroxymethylbilane synthase (HMBS, also known as porphobilinogen deaminase). Normally, the activity of this hepatic enzyme is low, so when in AIP, the activity falls further by about 50%, which limits the production of heme critically. It causes increased activity of 5-aminolevulinic acid synthase-1 (ALAS-1), which in turn causes increased levels of porphobilinogen, which can be one of the reasons for acute attacks of AIP [3]. Acute intermittent porphyria is one of the diseases where the complications are more life-threatening than the disease itself. Attacks may be identified by abdominal pain without peritoneal signs, associated with nausea, vomiting, hypertension, and tachycardia. These attacks can often get complicated by neurologic manifestations like mental changes, convulsions, and peripheral neuropathies, which can progress to respiratory failure/paralysis and hyponatremia [4]. Respiratory paralysis is the most dreaded complication, with a mortality rate of 90%, and is most commonly triggered by drugs [5].

Currently, AIP management consists of methods that decrease the formation of upstream metabolites of heme metabolism by effectively reducing the activity of the ALAS-1 enzyme. Prophylactic treatment includes terminating drugs causing porphyria and treating precipitating factors, but it is very limited and ineffective in terminating acute attacks. One of the treatments includes carbohydrate loading with glucose (10% dextrose), but carbohydrate loading is not effective for severe forms of the disease, and most patients end up with severe hyponatremia. Other treatments involve the off-label use of hemin infusions. They are successful in some patients, but with this, the patient requires frequent multiple infusions and a need for indwelling central venous catheter placement. This increases the risk for coagulopathy, phlebitis, thrombosis, and iron overload and may induce endogenous heme dependence [6]. Gonadotropin analogues are effective in certain female patients [7,8]. Liver transplantation would be the treatment of choice for individuals with severely compromised livers. Although potentially curative, it is not the treatment of choice because of the requirement of invasive surgery, lack of donors, and lifelong immunosuppression [9]. Therefore, currently, there is no modality that can treat or terminate an acute attack of acute intermittent porphyria.

Due to limitations in current therapeutic methods for AIP, there is a need for novel treatments that can terminate acute attacks with a reduction of symptoms and a minimal side effect profile. One such drug, givosiran, was recently approved by the FDA for treating acute intermittent porphyria. In this systematic review, we will determine the efficacy and side effect profile of givosiran and whether the benefits outweigh the risks of this treatment or not.

## Review

Methods

This systematic review was conducted based on the Preferred Reporting Items for Systematic Reviews and Meta-Analysis (PRISMA) 2020 guidelines [10].

Search Strategies

We searched databases like PubMed, PubMed Central (PMC), Medline, Cochrane Library, Internet Archive Scholar, Google Scholar, and Wiley Online Library. We used various combinations of 'Acute Intermittent Porphyria' and 'Givosiran' to search all databases. In PubMed, however, along with these keywords, the following strategy was developed and used to search relevant literature in PubMed's MeSH database: (("Porphyria, Acute Intermittent/drug therapy"[MeSH] OR "Porphyria, Acute Intermittent/therapy"[MeSH])) AND ("givosiran" [Supplementary Concept]). All the search strategies, the databases used, and the identified number of papers for each database are shown in Table 1.

**Table 1:** Number of papers identified in accordance of corresponding search strategies

No.	Search Strategy/ Keywords	Databases Used	No. of the Papers Identified
1.	Acute Intermittent Porphyria AND Givosiran	PubMed	38
2.	("Porphyria, Acute Intermittent/drug therapy"[Mesh] OR "Porphyria, Acute Intermittent/therapy"[Mesh]) AND ("givosiran" [Supplementary Concept])	PubMed	14
3.	Acute intermittent Porphyria AND Givosiran	Cochrane Library	34
4.	Acute intermittent Porphyria AND Givosiran	Internet Archive Scholar	91
5.	Acute Intermittent Porphyria AND Givosiran	Google Scholar	554
6.	Acute Intermittent Porphyria AND Givosiran	Wiley Online Library	64
	Total number of research papers identified	-	795
	Number of articles after removing duplicates	-	366

Eligibility Criteria

All the inclusion and exclusion criteria are mentioned in Table 2.

**Table 2:** Inclusion and exclusion criteria AIP: Acute intermittent porphyria

No.	Inclusion Criteria	Exclusion Criteria
1.	Papers written and published in the English language.	Gray literature and proposal papers.
2.	Papers focusing on AIP, Givosiran used for AIP, and treatment of AIP.	Papers involving non-human participants.
3.	Papers focusing on all age groups.	Papers in language other than English.
4.	Papers include Mixed types of Studies.	No full text is available.
5.	Papers are from all times.	Articles discussing aspects other than therapeutics for givosiran.
6.	Papers include full texts.	
7.	Papers involving human subjects.	

Selection Process

All articles were thoroughly checked, and all duplicate articles were removed. Each article was screened through titles and abstracts. In case of a conflict about eligibility, the concerns were discussed with all other co-authors and finalized by mutual consensus. The shortlisted articles were further evaluated by evaluating the full text, and only relevant articles were assessed. Only articles satisfying inclusion and exclusion criteria were shortlisted.

Quality Appraisal of the Studies

The shortlisted articles were checked for quality using relevant quality appraisal tools. Quality checks were done, and in case of any conflict, the problems were discussed with all other co-authors, and the final decision to include the paper was given by mutual consensus. A randomized control trial was assessed using the Cochrane Bias Assessment Tool. The Scale for Assessment of Narrative Review (SANRA Checklist) was used. Only studies that satisfied the quality appraisal were included in the systematic review. Tables 3, 4 comply the quality appraisal of only those studies that are included in the review.

**Table 3:** Cochrane bias assessment tool for clinical trials

Clinical Trials	The bias of the Randomization Process	Effect of Assignment on intervention	Effect of adhering to an Intervention	Bias due to Missing Outcome Data	Bias in the Measurement of Outcome	Bias in the Selection of Reported Result
Sardh E, et al. [11]	Low Risk	Low Risk	Low Risk	Low Risk	Medium Risk	Low Risk
Kuter D, et al. [12]	Low Risk	Low Risk	Low Risk	Medium Risk	Medium Risk	Low Risk
Balwani M, et al. [13]	Low Risk	Low Risk	Low Risk	Low Risk	Medium Risk	Low Risk

**Table 4:** Scale for assessment of narrative reviews

Reviews	Justification of the Article's Importance for Readership	Statement of Concrete/specific Aims or Formulation of Questions	Description of Literature Search	Referencing	Scientific Reasoning	Appropriate Presentation of Data
Zhao L, et al. [14]	2	1	0	2	2	1
Longo M, et al. [15]	2	1	0	2	2	1
Majeed CN, et al. [16]	2	2	0	2	2	1
Linenberger M, et al. [6]	2	1	0	2	2	0
Marcacci M, et al. [17]	2	1	0	2	2	1
Sardh E, et al. [18]	2	1	0	2	2	1
Bonkovsky H, et al. [19]	2	2	2	2	2	2
Thapar M, et al. [20]	2	2	2	2	2	2
Karl A, et al. [21]	2	2	2	2	2	2
Bonkovsky H, et al. [22]	2	2	2	2	2	2

Data Collection Process

After the articles were finalized for the systematic review and extracted, the primary outcomes were assessed along with other necessary information. Data was extracted from finalized articles and reviewed.

Results

Study Identification and Selection

**Figure 1:**
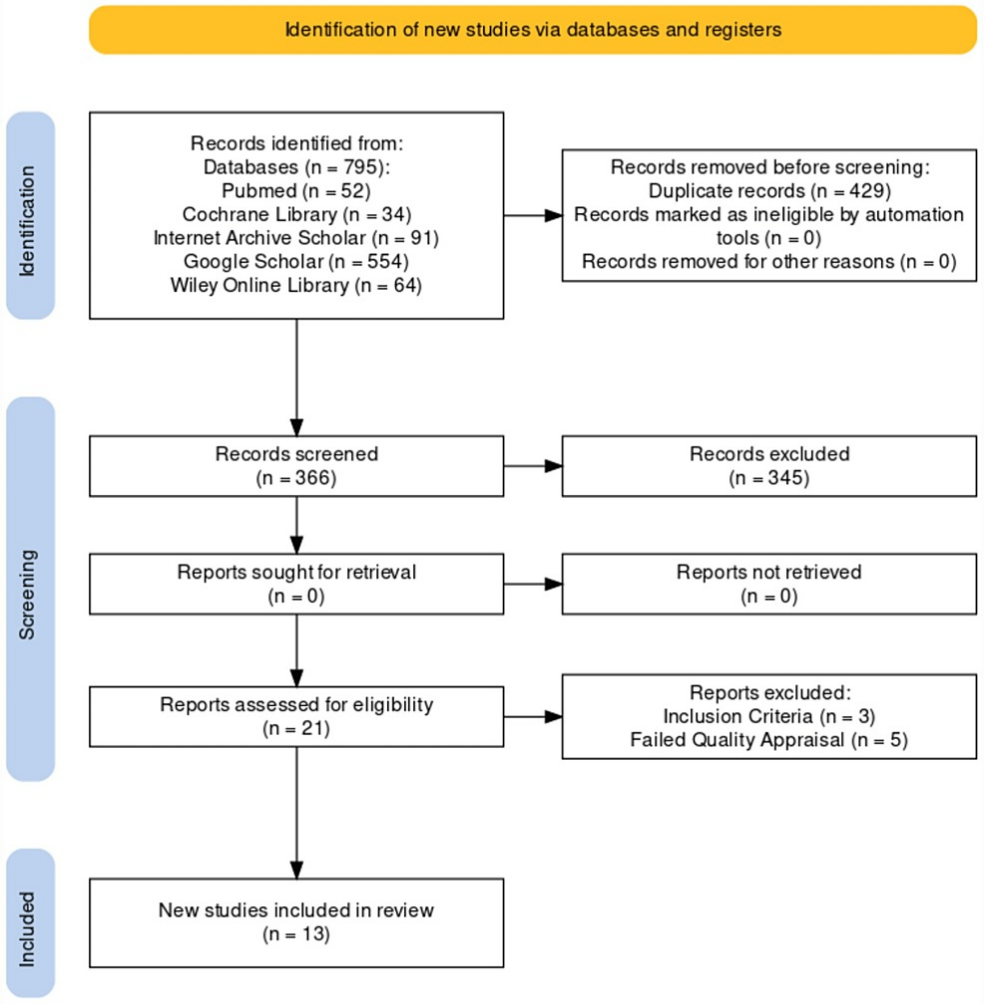
PRISMA flowchart PRISMA: Preferred reporting items for systematic reviews and meta-analysis

We gathered a total of 795 relevant studies using all the databases. Out of them, 429 duplicate studies were removed before the screening. After screening the remaining studies based on titles, abstracts, retrieving full text, and inclusion and exclusion criteria, we were left with a shortlist of 21 studies. These shortlisted full-text articles were used for quality appraisal, and eight more studies were removed. The remaining 13 studies were used for final review; three were randomized control trials, and 10 were reviews. The selection process for finalized studies is shown in Figure 1 of the PRISMA flowchart [10].

Detailed analyses of outcomes measured in relevant studies are shown in Table 5.

**Table 5:** Detailed analysis of relevant studies RCT: Randomized control trials, SC: Sub-cutaneous, AIP: Acute intermittent porphyria, AHP: Acute hepatic porphyria, ALA: Aminolevulinic acid, PBG: Porphobilinogen

Study	Type of Study	Population	Intervention	Comparing Group	Duration of Study	Outcomes Measured	Results	Follow-Up	Funding Sources	
Sardh E, et al. [11]	RCT	23; Patients without any recent attack of porphyria (no attacks in six months before baseline).	[A]: Single SC injection of increasing dose of givosiran (0.035, 0.10, 0.35, 1.0, or 2.5 mg/kg). [B]: Once monthly, one of the two doses of givosiran (0.35 or 1.0 mg/kg) for two months.	Placebo	[A]: Single SC injection. [B]: Total two injections 28 days apart.	1. Porphyria attack rates. 2. Urinary levels of δ-ALA and PBG.	It showed a significant improvement in the group taking Givosiran compared to the placebo.	No follow-up.	Alnylam Pharmaceuticals	
						1. Any severe adverse events.	It showed no significant difference in chances of having severe adverse events in people taking givosiran compared to placebo.			
		17; Patients with at least one prior attack of porphyria.	[C]: One of the two doses of givosiran (2.5 or 5.0 mg/kg) for four months.		[C]: Once monthly (total of 4 injections) or once quarterly (total of 2 injections) during 12 weeks.					
Balwani M, et al. [13]	RCT	94	Givorsiran 2.5 mg/kg given SC, monthly.	Placebo	Six months	The annualized rate of Porphyria Attacks in participants with AIP/AHP.	It showed a significant improvement in the group receiving Givosiran compared to the placebo.	Open-label Extension period was granted for the study.	Alnylam Pharmaceuticals	
						Annualized rate Hemin Administration.				
						Urine levels of Delta-ALA and PBG.				
						The weekly mean score of Daily worst pain.				
						The weekly mean score of daily nausea.	It was significantly higher in the group taking Givosiran compared to the placebo.			
						The weekly mean score of Daily Fatigue.				
						Injection site reaction.				

Discussion

Hemoglobin is a complex protein molecule composed of a prosthetic group in the form of heme and a protein, globin. Heme is a building block for many of the hemoproteins like hemoglobin, myoglobin, cytochromes, catalase, peroxidase, tryptophan pyrrolase, and nitric oxide synthase [22]. Although heme is synthesized by almost every tissue in the body, predominantly heme is synthesized in the mitochondria and cytoplasm of erythroblasts in the bone marrow (75%-80%) and hepatocytes in the liver (15%-20%) [3].

Biosynthesis of heme

**Figure 2:**
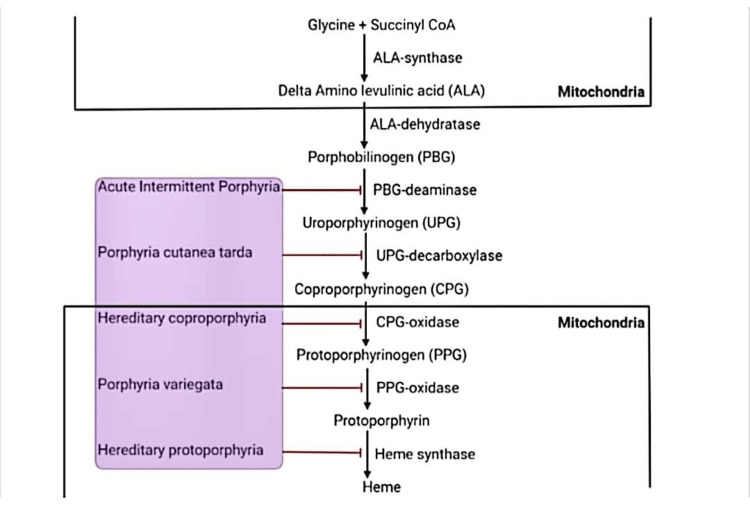
Pathways of heme synthesis CoA: Coenzyme A, ALA: Aminolevulinic acid, PBG: Porphobilinogen, UPG: Uroporphyrinogen, CPG: Coproporphyrinogen, PPG: Protoporphyrinogen. Created using BioRender.com

Heme biosynthesis involves seven steps with seven enzymes leading to the formation of a porphyrin ring incorporated with iron to form heme. Heme biosynthesis starts in the mitochondria with succinyl Coenzyme A (CoA) and glycine as substrates with the help of an enzyme called ALA synthase (ALAS) and pyridoxal phosphate as a coenzyme to form δ-amino levulinic acid (ALA). This is the only rate-limiting step in heme synthesis [3]. Subsequently, ALA is acted upon by enzymes like ALA dehydratase, PBG-deaminase, UPG-decarboxylase, CPG-oxidase, and PPG-oxidase to form a protoporphyrin ring. Lastly, the protoporphyrin ring is incorporated with ferrous iron by heme synthase to form heme [22]. The pathway of heme synthesis with its enzymes and pathology occurring from deficiency of the respective enzymes is shown in Figure 2.

An introduction to porphyria

Porphyria refers to a group of inborn errors of metabolism resulting from the mutation of genes responsible for the production of enzymes involved in heme biosynthesis. The word porphyria means 'purple' in Greek, from the color of the lesions seen. Porphyria is primarily a result of the accumulation of intermediates from the heme synthesis pathway into liver hepatocytes and erythroblasts of the bone marrow. Over-accumulation also leads to increased excretion of these precursors, which can cause end-organ damage. These are classified based on the site of their enzyme defect as hepatic, erythropoietic, and mixed. Acute intermittent porphyria is the most common of all porphyria [22,23].

Symptomatology of AIP

AIP constitutes a variety of symptoms, mainly neuropsychiatric, involving different systems and some long-term complications.

1) CNS symptoms include anxiety, confusion, depression, hallucinations, memory loss, and seizures. 2) PNS symptoms include neuropathic pain, sensory loss, respiratory failure, muscle weakness, and paralysis. 3) ANS symptoms include severe pain in the abdomen, chest, or back, hyponatremia, hypertension, tachycardia, nausea, vomiting, and constipation [23,24].

**Figure 3:**
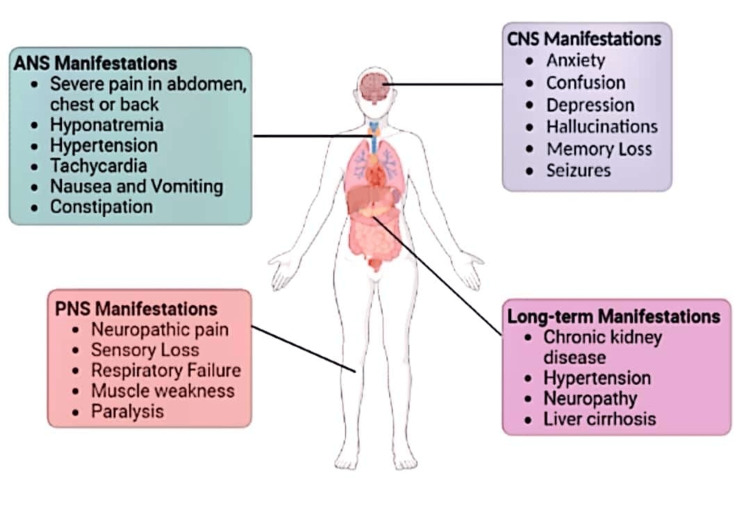
Symptomatology of acute intermittent porphyria ANS: Autonomic nervous system, PNS: Peripheral nervous system, CNS: Central nervous system. Created using BioRender.com

Symptoms of AIP affecting different systems are shown in Figure 3.

Treatment modalities

The primary management of AIP includes diagnosing an acute attack with the help of neurovisceral symptoms and laboratory tests and managing the symptoms while terminating the attack. It involves identifying and managing precipitating factors like treating occult infections and terminating porphyrinogenic medications. Other management methods involve suppressing the activity of the ALAS1 enzyme; this is done by carbohydrate loading and hemin infusions. As a last resort, liver transplantation can be used [6].

Hemin

For mild, moderate, and even severe cases of AIP where hospitalization is required, intravenous infusion of hemin (Normosang, heme-arginate in Europe; Panhematin, lyophilized hemin in the USA) is considered the first line. Hemin restores the heme pool in the hepatocytes and provides negative feedback on ALAS1 by downregulation transcription of ALAS1; it also decreases mitochondrial translocation of ALAS1 [24,25]. Although hemin has shown a very good response in terminating acute attacks of AIP, prophylactic heme therapy is off-label and should not be followed. Repeated regular hemin infusions have been shown to cause chronic hepatic inflammation and paradoxical upregulation of ALAS1 and Heme Oxygenase 1 (involved in heme catabolism), resulting in the recurrence of porphyria attacks, which remains a major setback for its use [26]. Moreover, hemin has shown effectiveness in treating acute symptoms like abdominal pain. Bonkovsky et al. showed that hemin infusion was a better pain-reduction alternative than other pain-reducing drugs like opiates, NSAIDS, sedatives, and beta-blockers. Still, hemin was unsuccessful in reducing the levels of plasma and urinary porphyrins [24-26,15]. Continuous hemin infusions can cause iron overload and raise ferritin levels, as 250 mg of heme-arginate contains approximately 22.7 mg of iron, thus increasing the risk for liver fibrosis, deposition of iron in the skin, and complications associated with it [27]. Moreover, continuous hemin infusions require the placement of a central venous catheter, which can lead to complications like coagulopathy, thrombosis, phlebitis, and infections [6].

Carbohydrate Loading

Low carbohydrate intake (less than 45%-60%) has been associated with an increased risk of disease severity in patients with AIP [28,29]. Although carbohydrate loading has not been proven as a therapeutic measure for AIP by controlled trials, dietary interventions in carbohydrate loading or intravenous glucose infusions have been proposed as a treatment for mild disease. In cases heme is unavailable, 300-500 g/day, preferably 10% dextrose in 0.45% saline, can be used as an initial treatment for managing AIP, but it also requires continuous glycemic monitoring to avoid any additional neurological complications [14,30]. It has been shown that glucose can inhibit hepatic ALAS1 transcription by stimulating insulin release by affecting peroxisome proliferator-activated receptor gamma coactivator1-alpha (PGC1-α) (called 'The Glucose Effect') and thereby causing porphyrin accumulation [14]. Moreover, glucose also provides substrates for heme biosynthesis [15].

Liver Transplant

Liver transplantation is used as a last resort for intractable acute diseases that are not responsive to medical management and are life-threatening. Orthotopic liver transplantation (OLT) can treat all the symptoms and is an effective treatment option. But just like every other transplant, the patient has to take an immunosuppressant for the rest of their life. Moreover, there is an increased risk of hepatic artery thrombosis in patients who undergo OLT for AIP [14]. Orthotopic LT may be the only curative therapy for AIP. Still, long waiting periods due to a low number of donors should be considered before considering OLT as a treatment option [15].

Givosiran: Therapy Using Small Interfering RNA

The concept of ribonucleic acid (RNA) interference revolutionized the treatment of various mammalian diseases. It follows an endogenous mechanism causing the degradation of specific mRNA. Small interfering RNA (siRNA) is a nucleic acid sequence that targets a specific mRNA and stops the translation, thereby preventing protein formation. This can theoretically target and downregulate almost all genes of interest [16]. Now the problem with this treatment modality is getting the siRNA to target a specific mRNA in a specific organ's cells.

Givosiran is a modified double-stranded small interfering ribonucleic acid (siRNA) comprised of 21 nucleotide sense strands and 23 nucleotide antisense strands, out of which the sense strand is attached to a triantennary N-acetylgalactosamine (GalNAc). This helps in the targeted molecule’s uptake by hepatic cells with the help of asialoglycoprotein receptors (ASGPR). Upon delivery into the liver, givosiran is incorporated into an RNA-induced silencing complex (RISC) and silences ALAS1 mRNA, thus halting the synthesis of ALAS1 protein. Givosiran, given subcutaneously, is absorbed with a peak plasma concentration after 0.5-5 hours. Elimination takes 4-10 hours due to the short half-life. Still, the effects of givosiran were seen for longer periods, in contrast to pharmacokinetics, which suggests that the effects of givosiran are driven by an active metabolite in the liver [18].

In a phase 1 trial (NTC02452372), 40 patients were given givosiran in different doses, out of which parts A and B consist of 23 patients without any severe attack in the past six months who were given either a single dose of 2.5 mg/kg givosiran or an increased dose of givosiran (0.035, 0.10, 0.35, 1.0, or 2.5 mg/kg) as shown in Table 5. It has been shown to effectively reduce overall annualized rates of porphyria attacks and reduce hemin use. In addition, it was also effective in reducing the levels of ALAS1 mRNA and urinary ALA and PBG levels by 96%, 86%, and 91%, respectively [11,14]. In part C, 17 patients with recurrent AIP attacks were given givosiran 2.5 or 5 mg/kg. It showed that it reduced ALAS1 hypersensitivity, normalized porphyrin levels with an acceptable safety profile and lowered the annual attack rate by 79% [11,15].

An open-label extension study (phases Ⅰ-Ⅱ) (NTC02949830) enrolled all the patients from phase Ⅰ, part C. Results of OLE showed that with a consistent 2.5 mg/kg dosing of givosiran, there was a decrease in urinary ALA and PBG of greater than equal to 80% at 12 months and >85% at 18 months. Annualized mean attack rates were reduced by 96%, and annualized hemin use was decreased by 98%, which continued to be at the reduced rate with continued dosing of givosiran [12,16,19,20].

Also, among the most reported adverse effects is homocysteinemia, which is very responsive to vitamin supplementation therapies [17]. Other adverse events like fatigue and nausea, which were calculated by a weekly mean score of daily fatigue and nausea and injection site reactions, elevations in liver transaminases, and pancreatic enzymes, were also reported in the open-label extension study [12,13,17,21]. Given the natural effects of the course of porphyria on the kidney, renal function was found to decline in a minority of patients with siRNA therapy. There was also a decline in liver function in a small population of patients. Hence, monitoring of liver function tests and renal function tests should be done in all patients taking therapy with givosiran [17].

Limitations

All identified studies were based on the limited number of clinical trials available. All the studies showed heterogeneity in sample size, measuring the variables, and the subjective nature of variables like pain, nausea, and fatigue. Not all the studies assessed all the similar variables and secondary outcomes. Also, functions were assessed in patients with normal renal and liver functions. Hence, patients with renal and liver impairment were excluded. Only papers in English were included in this review; hence, information from papers in languages other than English was not included.

## Conclusions

All the studies in this review showed that givosiran effectively reduces the recurrent attacks of AIP and, more importantly, terminates the recurrent attacks of porphyria. Moreover, givosiran also reduced the levels of ALAS1-mRNA in the blood level and reduced levels of PBG and ALA in the urine. Most of the studies showed that extended periods of use of givosiran (six months or more) could cause an increase in adverse events like nausea, fatigue, pain, injection site reactions, and, more importantly, renal and liver function impairment in a minority of patients, which was concluded in a few studies. Larger and more heterogeneous studies are required to assess the renal and liver function impairment caused by givosiran and give a better picture of its side effect profile. Also, studies should be done on the side effect profiles of givosiran in volunteers patients with pre-existing renal or liver impairment.
